# Androgen-Dependent Prostate Cancer Cells Reprogram Their Metabolic Signature upon GLUT1 Upregulation by Manganese Superoxide Dismutase

**DOI:** 10.3390/antiox11020313

**Published:** 2022-02-04

**Authors:** Isabel Quiros-Gonzalez, Pedro Gonzalez-Menendez, Juan C. Mayo, David Hevia, Francisco Artime-Naveda, Sheila Fernandez-Vega, Mario Fernandez-Fernandez, Pablo Rodriguez-Gonzalez, José I. Garcia-Alonso, Rosa M. Sainz

**Affiliations:** 1 Departamento de Morfologia y Biologia Celular, Instituto Universitario de Oncologia del Principado de Asturias (IUOPA), University of Oviedo, 33006 Oviedo, Spain; mayojuan@uniovi.es (J.C.M.); heviadavid@uniovi.es (D.H.); artimefrancisco@uniovi.es (F.A.-N.); fernandezsheila@uniovi.es (S.F.-V.); 2 Instituto de Investigación Sanitaria del Principado de Asturias (ISPA), 33001 Oviedo, Spain; rodriguezpablo@uniovi.es (P.R.-G.); jiga@uniovi.es (J.I.G.-A.); 3 Institute de Genetique Moleculaire de Montpellier, University of Montpellier, CNRS, 34293 Montpellier, France; pedro.gonzalez-menendez@igmm.cnrs.fr; 4 Departamento de Quimica Fisica y Analítica, Universidad de Oviedo, Julián Clavería 8, 33006 Oviedo, Spain; fernandezmario@uniovi.es

**Keywords:** prostate cancer, metabolism, redox, SOD2, GLUT-1, SDH

## Abstract

Prostate cancer is the second leading cause of cancer in men across the globe. The prostate gland accounts for some unique glycolytic metabolic characteristics, which causes the metabolic features of prostate tumor initiation and progression to remain poorly characterized. The mitochondrial superoxide dismutase (SOD2) is one of the major redox metabolism regulators. This study points out SOD2 as one major regulator for both redox and glycolytic metabolism in prostate cancer. SOD2 overexpression increases glucose transporter GLUT-1 and glucose uptake. This is not an insulin-mediated effect and seems to be sex-dependent, being present in male mice only. This event concurs with a series of substantial metabolic rearrangements at cytoplasmic and mitochondrial level. A concomitant decrease in glycolytic and pentose phosphate activity, and an increase in electron transfer in the mitochondrial electronic chain, were observed. The Krebs Cycle is altered to produce amino-acid intermediates by decreasing succinate dehydrogenase. This in turn generates a 13-fold increase in the oncometabolite succinate. The protein energy sensor AMPK is decreased at basal and phosphorylated levels in response to glucose deprivation. Finally, preliminary results in prostate cancer patients indicate that glandular areas presenting high levels of SOD2 show a very strong correlation with GLUT-1 protein levels (R^2^ = 0.287 *p*-value < 0.0001), indicating that in patients there may exist an analogous phenomenon to those observed in cell culture and mice.

## 1. Introduction

Prostate cancer is the main type of male cancer and the third cause of cancer-related male deaths in the USA and Europe [1]. Aging is the main risk factor, and therefore, age-related alterations such as redox metabolism have been proposed as one of the leading drivers. Most of the cases are sporadic and lacking a clear group of driver genes for the disease [2].

Prostate cancer is unique in its metabolism [3,4]. Prostate epithelial cells produce high levels of citrate by inhibiting the Krebs Cycle (KC), which in turn promotes the activation of the glycolytic metabolism. However, there is still a “metabolic switch” during prostate tumor progression promoting Oxidative Phosphorylation (OXPHOS) [3]. This so-called “reverse Warburg” leaves prostate cancer in an unknown territory from a metabolic point of view. A remarkable point is the fact that diabetes, contrary to other tumor types, plays a protective role in prostate cancer incidence and progression [5].

Manganese-dependent superoxide dismutase (SOD2) is a mitochondrial enzyme which scavenges the main radical produced during OXPHOS, the superoxide radical O_2_^λ−^. The strategic location of this protein in the mitochondrial matrix is essential for survival [6,7]. However, beyond a minimum expression, it varies a lot as it is easily regulated in a time- and place-specific manner [8]. Often considered as an antioxidant enzyme, it is rather a redox modulator as it produces H_2_O_2_ as a second messenger. H_2_O_2_ increase due to SOD2 overexpression has been linked to differentiation [9,10], anti-tumor therapy resistance, and metastasis in the prostate [8]. Although most of the studies are focused on the impact of Sod2 polymorphisms in tumor incidence and progression [11], emerging evidence points to its role on specific hallmarks of cancer progression such as hormone independence and metabolic spread in the prostate and breast [12,13]. Considering its essential role in mitochondrial homeostasis, it is not surprising that very recent reports have pointed out its role in metabolism regulation [11,12,13]. One of the most interesting aspects is the ability to reduce androgen signaling [9]. Androgens are essential to keep the tissue homeostasis in the prostate, and disarrangements in androgen signaling including receptor overexpression and splicing variants have a strong impact on prostate cancer progression [14], metabolism [15], and redox profile [16].

The glucose transporter solute carrier family 2 facilitated glucose transporter member 1, also known as GLUT-1, is the major transporter in the organism, independent of insulin regulation, and is present in all the tissues. GLUT-1 is related to tumor progression for different tumor types [17]. In prostate cancer, GLUT-1 expression is higher in tumor than in non-tumor tissue but also correlates with tumor progression [18].

This report shows the effect that the sole overexpression of SOD2 has in the metabolic profile of androgen-dependent prostate cancer affecting glycolytic and KC through succinate dehydrogenase regulation. The main switch of this metabolic rearrangement is the upregulation of the glucose trans-porter GLUT-1. We provide evidence of these relations in mouse and human prostate cancer. 

## 2. Materials and Methods

### 2.1. Cell Culture

LNCaP cell line was purchased from ATCC (CRL-1740). MOCK and SOD2 clones were established by transfection of the pcDNA3.0 and pcDNA3.0-Sod2 kindly provided by Dr St. Clair (University of Kentucky, Lexington, KY, USA), with further selection using 500 μg/mL of G418 (Sigma-Aldrich) in the culture media for 2 months. The maintenance and experimental cell culture conditions were RPMI1640 + 10% FBS and anti-biotic, anti-mycotic cocktail (Thermo, Madrid, Spain).

For glucose deprivation assay, glucose concentration in control groups was always set at 2 g/L (11 mM). Glucose deprivation was achieved by using complete medium without glucose (Lonza, Celta Ingenieros, A Coruña, Spain) and cells were kept on these media for 48 h for cell death assays and 24 h for western blot.

### 2.2. Enzymatic Activity

In 96-well plates, 190 µL of assay mixture, 5 µL of sample (17 μg protein), and 5 µL of xanthine oxidase were consecutively added and the cytochrome c oxidation absorbance at 550 nm was measured after 4 min. 

Each sample was run in triplicate and one reaction was carried out only with xanthine and xanthine oxidase to calculate the oxidation slope without inhibition (non-inhibition slope). We consider an enzyme unit the activity able to reduce the oxidation slope a 50%. Finally, the enzyme unit (U) was relativized against the amount of protein (U/µg prot).

### 2.3. H_2_O_2_ Quantification

Hydrogen peroxide was determined by amperometric detection using a portable potentiostat (µSTAT200, DropSens, Asturias Spain). To this aim, disposable screen-printed carbon SPCE doped with phthalocyanine was used (410, Metrohm DropSens). The potential applied was 0.4 V in an interval of 2 min with a measure acquisition of 0.25 s. A 35 µL measure of each culture medium was used. Hydrogen peroxide standards in PBS of 1, 2.5, 5, and 10 µM concentration were prepared as calibration.

### 2.4. Western Blot

Cells were seeded at an initial density of 5 × 10^4^ cells/mL and allowed to attach for 48 h. Treatments are detailed bellow (see Glucose deprivation). Cell pellets were lysed with Tri-Detergent lysis buffer (0.5% deoxicolate, 1% Igepal, and 0.1% SDS). For GLUT-1, protein extracts were not frozen before western blot. Protein extracts were loaded for electrophoresis and then wet transfer into a PVDF membrane (Millipore, Merk, Madrid, Spain). Primary antibodies were used as follows: SOD2 (Millipore, 1:5000), GLUT-1 (1:2500 Millipore 07-1401), SDH (1:1000 Sigma-Aldrich HPA002868), PAMPK^thr172^ (1:1000 Millipore 07-626), AMPK (1:1000, Millipore 07-350), and Actin (1:10,000 SantaCruz, sc-69879). Secondary antibodies were conjugated with HRP and ECL (Millipore) was used as substrate for chemoluminescence. Images were processed with Fiji and results were normalized against actin and expressed as arbitrary units.

### 2.5. Measure of Metabolic Flux Profile

Metabolic profiling of cells was undertaken using a Seahorse XFe24 Analyzer in XFe24 microplates.

Cells were seeded by suspension in the growth medium described above in the apart cell culture in a Seahorse XF24 Cell Culture Microplate (Agilent, Madrid, Spain 100777-004) and placed in the cell culture incubator for three hours to adhere. After cells attached, 150 µL of growth medium was added to each well. The cells were allowed to stand overnight in the cell incubator at 37 °C. The next day, the growth medium was replaced by Seahorse XF RPMI pH = 7.4 (Agilent, 103576-100), and either Real-Time ATP Rate Assay (Agilent, 103592-100) or Glycolytic Rate Assay (Agilent, 103344-100) was performed according to the manufacturer’s instructions.

### 2.6. Glucose-Uptake Assay

Cell were washed twice with RPMI 1640 complete medium without glucose and incubated in media containing 2 mM 2-deoxy-D-glucose (2DG) for 20 min. Cells were lysed with 0.1 M NaOH and graded heating from 60 °C to 85 °C for 60 min. After that, lysates were neutralized with 0.1 M HCl and 200 mM triethanolamine. The 2DG was measured after 45 min incubation with assay solution (50 mM TEA, 50 mM KCl, 0.02%BSA, 0.1 mM NADP^+^, 0.2 U/mL diaphorase, 6 mM resazurin sodium salt, and 20 U/mL glucose-6-phosphate dehydrogenase). Fluorescence was measured in a Sinergy H4 hybrid reader (ex 550 nm, em 605 nm).

### 2.7. Pentose Phosphate Assay

Cells were incubated with 1/3 volume of 1 mM tetranitroblue tetrazolium, 0.5 mM glucose-6-phosphate, and 0.5 mM NADP+ for 4 h. The development of purple color was measured spectrophotometrically (570 nm). 

### 2.8. Animal Experimental Procedures

*Sod2*^+/++^ mice were kindly provided by Dr. Robia Pautler (Baylor College of Medicine, Houston, TX, USA). They were bred in a C57BL6/J genetic background, carrying a human *Sod2* transgene under the transcriptional control of the human β-actin promoter [19]. Mice were maintained under controlled environmental conditions (12:12 light:dark) with ad libitum access to food and drinking water. Experiments and procedures were conducted in accordance with European Directive 2012/63/UE and were approved by the Ethical Committee Board for Animal Experiments at the University of Oviedo (Experimental protocol PROAE 03/2016)

To measure glucose tolerance, 18-week-old mice fasted overnight (*n*^male^ = 5, *n*^female^ = 3) received an intraperitoneal injection of glucose (2 mg/g body weight) and blood glucose was determined after 15, 30, 60, and 120 min from the tail vein using a One Touch Ultra EasyTM glucometer (Johnson&Johnson). 

Insulin levels were measured in the plasma of overnight-fasted mice. Blood was extracted postmortem, collected in EDTA-treated tubes, and immediately centrifuged at 3000× *g* for 10 min at RT. Samples were frozen at −80 °C until analysis. Insulin levels were measured by ELISA following the manufacturer’s instructions (Millipore, catalogue no. EZRMI-13K).

### 2.9. Metabolomic Assay

Cell were grown in media supplemented with 2 g/L of D-glucose-^13^C6 for 24 h. The intracellular metabolites were obtained from pelleted cells after extraction with 100% methanol at −80 °C, following a double extraction with milli-Q water. Metabolites were dissolved in 100 µL of 2% methoxyamine hydrochloride in pyridine and incubated at 40 °C for 8 min on a compact thermomixer. Next, 150 µL of N-tert-butyldimethylsilyl-N-methyltrifluoroacetamide plus 1% tert-butyldimethylchlorosilane was added and samples were incubated for 10 min at 60 °C. Derivatized samples were centrifuged at 14,000× *g* for 2 min to remove debris. Clear samples were transferred into GC vials for GC–MS analysis. A gas chromatograph Agilent 7890 coupled to a triple quadrupole mass spectrometer Agilent 7000 Series Triple Quad GC/MS (Agilent) was employed, operating in selected-ion monitoring mode at 70 eV. For the measurement of the mass isotopomer distribution of the intracellular metabolites, the experimental isotopic distribution measured by GC–MS was assumed to be a linear combination of all possible incorporation of ^13^C in the molecule (isotopomers). The relative contribution of isotope patterns in the experimental mass spectra was calculated by multiple linear regression. Heatmap and clustering 181 analysis were performed using the heatmap3 add-in package with R project open-source 182 software [20]. Enrichment and pathway analyses were performed using MetaboAnalyst [21]. 

### 2.10. Mitotracker Staining

Cells were seeded at an initial density of 2.500 cell per well in 24-well plates. Cells were stained and fixed following manufacturer’s information (staining time 30 min). At least three random fields were analyzed per triplicate of each cell line. Image J was used to measure staining intensity and one R.O.I. was drawn per cell.

### 2.11. Flow Cytometry: Cell-Cycle Analysis and Annexin V Staining

For cell-cycle analysis, cells were fixed in 70% ethanol and then incubated in PBS supplemented with glucose (1 g/L), RNase (100 U/mL), and 50 mg/mL propidium iodide (PI) for 30 min at 37 °C. For viability assays, cells were incubated with 1 mg/mL PI for 10 min at RT. Samples were analyzed by FACS using a Cytomics FC500. 

### 2.12. MTT

Cells were seeded in 96-well plates. After the corresponding treatment, 50 mg MTT (from a 5 mg/mL stock) was added to each well for 4 h. Then, a volume of lysis buffer (20% SDS and 50% dimethylformamide) was added and incubated at 37 °C overnight. Absorbance was measured at 570 nm vs. 690 nm in a Varian Cary 50-MPR UV-Vis spectrophotometer (Agilent). Results are shown as the mean of six samples ± SEM.

### 2.13. Human Prostate Samples

Samples were obtained from radical prostatectomies preformed in prostate cancer patients (*n* = 5) at the Central Hospital of the Principality of Asturias between 2000 and 2009. Formalin-fixed paraffin-embedded (FFPE) samples were provided by the Principado de Asturias BioBank (PT17/0015/0023), integrated in the Spanish National Biobanks Network, and histological diagnosis was confirmed by an experienced pathologist. Samples were processed following standard operating procedures with the appropriate approval of the Ethical and Scientific Committees of the Hospital Universitario Central de Asturias and the Regional CEIm from Principado de Asturias. Patient data are summarized in Suppl. Table S1. The patients had not received any anti-androgenic treatment prior to the surgery.

### 2.14. Immunohistochemistry and Immunocytochemistry

Prostate adenocarcinoma sections (5 μm) were upon antigen retrieval (citrate 10 mM, pH 6.1) for 10 min and then blocked to remove unspecific signal (1.5% Goat Serum in PBS-0.15% Tween-20). Slides were incubated overnight on GLUT-1 (1:200), and, after washing (3×), incubated with the secondary antibody (Fab fragment rabbit phycoerythrin (1:100) for 1 h. After washing (3×), the solution for SOD2 antibody (Millipore 1:200) was added O.N. After washing (3×), Alexa 488 anti-rabbit solution (1:300) was added for 1 h. Washing, mounting, and DAPI counterstaining were performed following standard protocols.

LNCaP-MOCK and LNCaP-SOD2 cells were seeded onto treated coverslips (Thermanox^TM^), three for each cell type. Cells were fixed using 4% PFA, 4 °C overnight. Permeabilization and blockage was performed by using 3% BSA-0.1% Tween for 30 min. Coverslips were incubated overnight with GLUT-1 primary antibody (1.100) and F’ab fragment Rabbit Phycoerythrin (1:200) was used as secondary antibody. 

Micrographs were taken at a final magnification of 400× and 630× and seven random fields were captured. Cell contour was used to delimitate one R.O.I. per cell, and fluorescence intensity per cell was measured. Fiji was used as image software.

### 2.15. Statistical Analysis

Prism software was used for statistical analysis and graph representation. Normality was assessed by Shapiro–Wilk test for small sample size (*n* = 3) and Kolmogorov–Smirnov for bigger sample sizes. For statistical analysis between two groups, the *t*-test was used. For multiple comparisons, one-way ANOVA was used. Simple linear regression analysis was performed for correlation. For all panels, values are expressed as Mean ± SEM; * = *p*-value < 0.05, ** = *p*-value < 0.01, *** = *p*-value < 0.001, **** = *p*-value < 0.0001.

## 3. Results

### 3.1. Stable SOD2 Overexpression Increases GLUT-1 Levels and Glucose Uptake, Altering Glycolysis and OXPHOS

In order to increase the levels of SOD2, we transfected an *SOD2*-bearing plasmid and selected a stable cell line in the androgen-dependent LNCaP cell line. The stable expression of the *SOD2* produced five-fold protein overproduction compared with the native cell line (Suppl. Figure S1B). This concurs with the expected increase in the enzymatic activity on the verge of significance (Suppl. Figure S1C, *p*-value = 0.0625) and a modest increase in the extracellular H_2_O_2_ (Suppl. Figure S1D). No differences in proliferation measured by cell-cycle phase distribution were observed (Suppl. Figure S1E).

**Figure 1:**
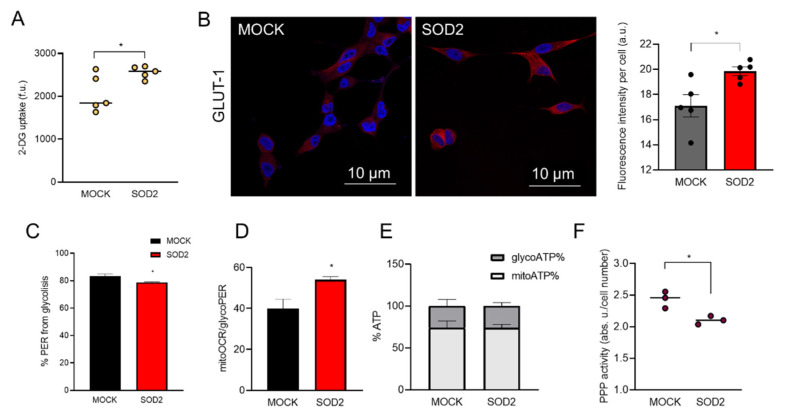
SOD2 increases glucose uptake and GLUT-1 protein levels. (**A**) 2-deoxy-D-glucose uptake in LNCaP-MOCK (MOCK) and LNCaP-SOD2 (SOD2) cells. (**B**) Micrograph 400× magnification of Glut-1 (red) IHC in LNCaP cells, DAPI (blue is shown as counterstaining). (**C**–**E**) Seahorse metabolic analysis of MOCK and SOD2 cells, (**C**) percentages of proton efflux rate from glycolysis (glycoPER), (**D**) ratio of oxygen consumption rate in mitochondria (mitoOCR) over glycoPER; (**E**) ATP produced during glycolysis (glycoATP) and OXPHOS (mitoATP). Data are expressed on percentage. (**F**) Pentose Phosphate Pathway activity (PPP) in MOCK and SOD2 cells. (**B**–**F**) *n* = 3 all groups. For all panels, values are expressed as Mean ± SEM; * = *p*-value < 0.05, *t*-test.

Based on previous reports [11,12,13], we expected SOD2 protein expression to have re-arrange cell metabolism. To determine the general metabolic status of the SOD2-overexpressing cells we first measured 2-deoxy-D-glucose uptake. The increase in SOD2 favors glucose uptake (Figure 1A). Then, we examined the levels of glucose transporter GLUT-1, the major importer of glucose intake that could be responsible uptake results, and we found an increase in the production of the glucose transporter GLUT-1 in SOD2-overexpressing cells (Figure 1B). Interestingly, this increase is not simultaneous with an increase in glycolysis, measured by Proton Efflux Rate from the glycolysis (glycoPER), that shows a slight but significant decrease (Figure 1C). Consequently, the ratio mitoOCR/ glycoPER) used to evaluate the OXPHOS vs. glycolysis metabolism indicates that SOD2-overexpressing cells show a higher contribution from OXPHOS (MOCK = 0.40 ± 0.02 vs. SOD2 = 0.54 ± 0.01; Figure 1C). However, the increase in the ratio mitoOCR/glycoPER does not produce an increase in the ATP coming from mitochondrial origin (Figure 1E). Our results indicated that glucose increase was not destinated to glycolysis or Pentose Phosphate Pathway (PPP) (Figure 1F), which showed a significant decrease in SOD2 cells. To discard some deleterious effect of the mitochondrial H_2_O_2_, we count the average number of mitochondria per cell, finding that is similar in both cell types (Suppl. Figure S2). 

### 3.2. SOD2 Increases Glucose Uptake in Mice

**Figure 2:**
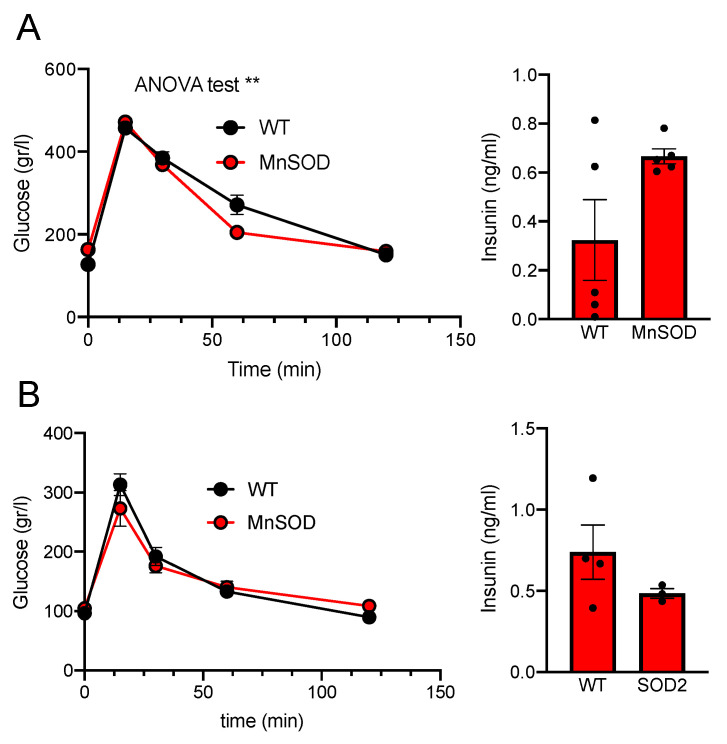
Glucose Tolerance Test on WT and SOD2 transgenic mice, (**A**) males, (**B**) females. Glucose (2 mg/g body weight) was injected IP. Basal insulin levels (Ins) are expressed in ng/mL. n_males_ = 5; n_females_ = 4. For all panels, values are expressed as Mean ± SEM; ** = *p*-value < 0.01, ANOVA test.

Analogously to the results obtained in LNCaP, we studied the rate of glucose uptake in *SOD2* transgenic mice. These mice have an additional copy of *Sod2* and show faster glucose uptake in a glucose tolerance test (GTT) than WT (ANOVA *p*-value = 0.0067; Figure 2A, left). However, there was no significant increase in insulin secretion that could be responsible of this uptake (WT = 0.32 ± 0.16 vs. SOD2 = 0.66 ± 0.07; Figure 2A right). This response seems to be unique in males, as females overexpressing SOD2 showed no difference in GTT (Figure 2B).

### 3.3. Prostate Adenocarcinoma Cells Overexpressing SOD2 Present a Metabolic Rearrangement of Krebs Cycle and Aminoacidic Pathways

**Figure 3:**
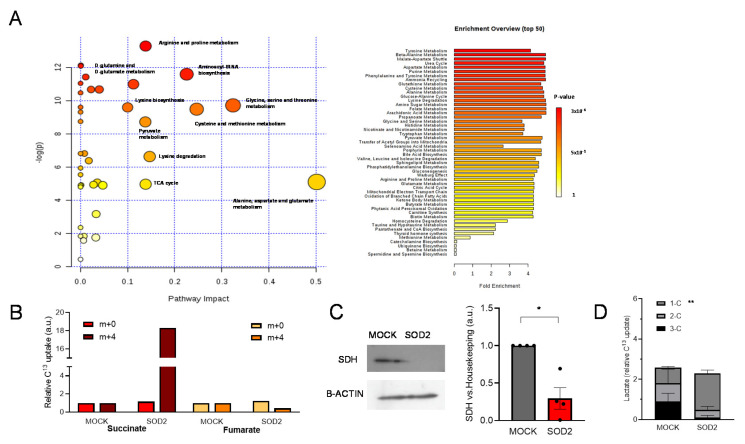
SOD2-overexpressing cells showed an increase in amino-acid synthesis pathways and a partial Krebs Cycle blockage through a decrease in succinate dehydrogenase. Metabolomic analysis of LNCaP-Mock (MOCK) and LNCaP-SOD2 (SOD2). (**A**) Summary graph and list of the main metabolic pathways altered in SOD2 cells, showing impact and enrichment. (**B**) Relative uptake of ^13^C by succinate and malate, indicating accumulation of recently formed succinate (m + 4) and a decrease in fumarate. (**C**) Protein levels of succinate dehydrogenase (SDH) in MOCK and SOD2 cells, values of densitometric quantification relative to B-ACTIN are shown. (**D**) Levels of lactate loaded with 1-, 2-, or 3-^13^C in its structure (*n* = 3 each group). For all panels, values are expressed as mean ± SEM; *t*-test was used as statistical analysis; * = *p*-value < 0.05, ** = *p*-value < 0.01.

As we illustrated in Figure 1, the increase in internalized glucose did not increase either energy production in glycolysis or OXPHOS or increase PPP activity. In order to unveil the destination of the glucose uptaken, we analyzed the metabolomic flux of these cells using isotopically labeled ^13^C-glucose. SOD2 overexpressing cells showed an increase in the amino-acid pathways, alanine, aspartate, glutamate, glycine, serine, and threonine (Figure 3A). Succinate showed a significant accumulation (18-times increase) and a subsequent decrease in the downstream metabolite on the KC, fumarate, was also observed (Figure 3B). 

The KC enzyme which converts succinate into fumarate is the succinate dehydrogenase (SDH). This enzyme has a double role linking the KC with the OXPHOS as it is also part of the Complex II of the respiratory chain. Consequently, with the accumulation of succinate, we observed a three-fold decrease in SDH in SOD2 cells (Figure 3C) as well as a substantial decrease in mitochondria Complex II activity (Suppl. Figure S3).

Among the labeled carbon species, the proportion of labeled lactate (Figure 3D) is noteworthy. The levels of 1-C were significantly increased in SOD2-overexpressing cells with a not-significant, yet strong, decrease in 3C lactate (MOCK = 0.90 ± 0.40 vs. SOD2 = 0.11 ± 0.09; *p*-value = 0.13). In order to generate 1-C pyruvate and then 1-C lactate, 3-labeled pyruvate has to cycle twice KC. This indicates that KC cycles faster in SOD2 cells despite the low levels of SDH.

### 3.4. Survival to Glucose Deprivation Is Ameliorated by SOD2 Overexpression

**Figure 4:**
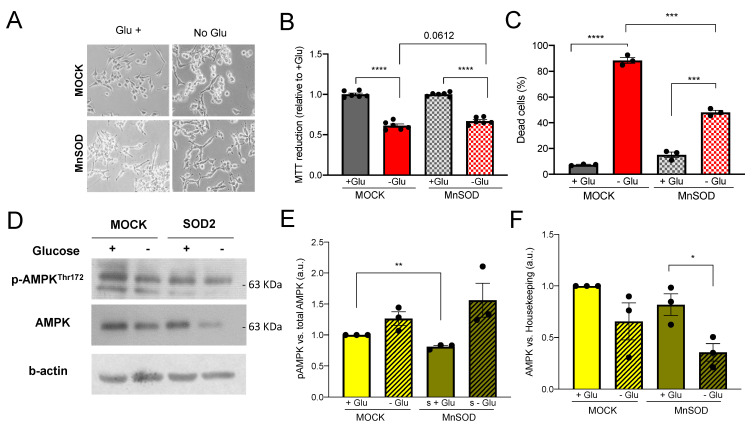
SOD2-overexpressing cells are more resistant to Glucose deprivation and total AMPK protein levels are toned down. Glucose deprivation response in LNCaP-MOCK (MOCK) and LNCaP-SOD2 (SOD2). (**A**) Representative micrographs of morphological changes upon glucose deprivation, magnification 100×. (**B**) Cell death measured by Annexin V flow cytometry analysis. (**C**) Cell viability measured by MTT mitochondrial reduction; the values are represented as % for each Glu+ control. (**D**–**F**) Protein levels of phosphorylated (**D**,**E**) and total-AMPK (**D**,**F**) in MOCK and SOD2 cells, values for densitometric quantification relative to B-ACTIN are shown. For all panels, values are expressed as mean ± SEM; *t*-test was used as statistical analysis; * = *p*-value < 0.05, ** = *p*-value < 0.01, *** = *p*-value < 0.001, **** = *p*-value < 0.0001.

Glucose (Glu) import through GLUT transporters is essential for cell survival. SOD2-overexpressing cells have an increase in glucose uptake, GLUT-1 availability, and OXPHOS electron transfer, producing an increase in amino-acid synthesis rather than energy production. In this context, we analyzed the effect of Glu deprivation on cell survival (Figure 4A). 

When we observed cell viability using MTT, which also illustrates mitochondrial activity, we saw a trend on the verge of significance (*p*-value = 0.067) between MOCK and SOD2 in MTT reduction in absence of Glu, suggesting SOD2 cells were more resistant to glucose-deprivation-induced cell death (Figure 4B). This trend was confirmed by annexin V results. After 48 h of deprivation, 88.47% of MOCK cells were dead, while this percentage decreased to 48.15% in SOD2-overexpressing cells (Figure 4C). This is supported by results of our own group which prove that cell death induced by glucose deprivation is triggered by oxidative stress [22]. MTT results might confirm a decrease in mitochondrial activity in SOD2-overexpressing cells that counterbalances the increased survival as it was also shown in Suppl. Figure S3.

We next studied AMPK levels. AMPK is the major sensor of cell energy status, which is regulated by SOD2 [23]. We observed differences at both total and phosphorylated protein levels. The basal phosphorylation of PAMPK is significantly lower (Figure 4D,E) and, interestingly, in absence of Glu, total levels of AMPK drop, showing a 2.3-fold decrease in SOD2 cells (Figure 4D,F). 

### 3.5. GLUT-1 and SOD2 Levels Correlate Spatially in Human Prostate Cancer

**Figure 5:**
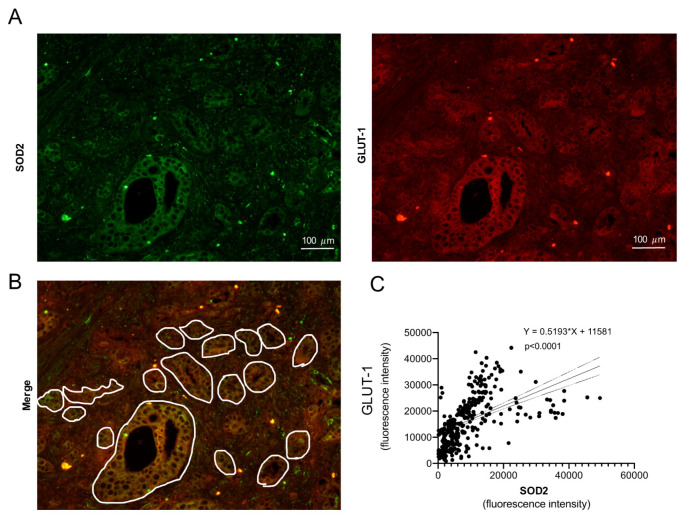
SOD2 and GLUT-1 protein levels show high correlation in prostate adenocarcinoma patient samples. Human prostate cancer sample micrographs (magnification 100×) were analyzed for the expression pattern of SOD2 (**A**, green) and GLUT-1 (**A**, red) proteins by using double IHF. Only glandular area was selected (**B**) and the intensity of the signal for both proteins quantified. (**C**) Correlation plot for the total number of glands quantified in all the patients (*n* = 5); correlation equation and *p*-value are shown.

The relationship between Glut-1 and SOD2 is more relevant if it can be replicated in human prostatic tissue. Samples from prostate cancer patients were analyzed for SOD2 and GLUT-1 protein expression (Figure 5A). In order to choose an analogy with the in vitro model, only the glandular area was considered for this study (Figure 5B). A significant positive correlation (≤0.0001, R^2^ = 0.287) was found on the five patients, either analyzed individually (see Suppl. Figure S4) or collectively (Figure 5C), showing that high SOD2 protein levels overlap with high GLUT-1 protein levels.

## 4. Discussion

This manuscript presents for the first time how physiological variations of SOD2 protein can reprogram prostate metabolism through GLUT-1 and SDH affecting glucose uptake, KC, and mitochondrial function. Recent reports have proposed the role of SOD2 and its substrates and products in metabolism [23,24]. Considering the central role of this protein in mitochondrial homeostasis, it is expected a direct influence in OXPHOS. However, our work shows a much broader regulation. 

Upon glucose, at membrane level, the effect is by increasing the GLUT-1 protein and glucose uptake. This is the first time that SOD2 has been shown to regulate GLUT transporters. Importantly, this regulation seems to be present in prostate cancer patients, as we have observed a very strong correlation (*p*-value below 0.0001) between SOD2 and GLUT-1 protein in prostate cancer samples from patients, indicating that wherever SOD2 is high, so is GLUT-1. 

These results might contrast with those from Zhou [25], where downregulation of SOD2 was associated with an increase in glucose uptake in colon cancer cells. In this case, the metabolism of the prostate tissue presents a unique scenario that may account for this difference. 

The increased uptake is also observed in mice triploids for *Sod2*. Normal systemic glucose metabolism does not seem to be responsible for the faster glucose uptake in mice as insulin levels do not differ significantly. Interestingly, both features are only observed in male mice. This could indicate that the SOD2-dependent uptake could be regulated by androgens or up-taken at the prostate tissue. 

Although androgen-dependent prostate cancer cells LNCaP that overexpress SOD2 were able to uptake more glucose, we did not find any increase in glycolytic metabolism. In fact, we observed a decrease in PER from glycolysis and no changes in ATP from glycolysis.

The next level of regulation is matrix mitochondria. In androgen-dependent prostate cancer cells overexpressing SOD2, metabolomics revealed an increase in amino-acid intermediates to provide the bricks that are needed to increase proliferation. Similar phenomena have been seen in prostate cancer upon redox [23] and androgen–mitochondrial regulation [26]. On the latter, androgen-dependent cells have an androgen-dependent activation of the KC through the MCP2 (Mitochondrial Pyruvate carrier 2), and in absence of androgens they switch to a glutamine-dependent KC metabolism. Our group has previously shown that SOD2 might maintain prostate cancer survival in absence of androgens [9], and also substantial differences in tumor oxygenation rate between androgen-dependent and -independent prostate cancers have been described [27]. Our current data propose a new pathway that would allow an androgen-independence transition independently of those metabolic changes occurring in absence of androgens. 

Our results indicate that downregulation of AMPK might be responsible for this reprogramming. A downregulation of AMPK pathway has been described before as responsible for a similar reprogramming [28]. We find that basal PAMPK is decreased in SOD2 cells; however, the phosphorylation levels in response to glucose deprivation reach the same levels than Mock, indicating that the pathway is fully functional. Additionally, total AMPK protein in the presence of glucose is reduced. Our hypothesis is that SOD2 partially tunes down the AMPK signaling pathway, and this contributes to improving the survival. This might disagree with other reports [23], however, our model uses a subtler modulation of SOD2_redox metabolism than the one observed in Hart et al. but also more likely to be found in nature. 

This massive amino-acid pathway reprogramming occurs through alterations in KC. Our results clearly point out a downregulation of succinate dehydrogenase (SDH) as the main event of this reprogramming. The downregulation partially blocks the transit of the cycle with the subsequent accumulation of succinate and decrease in malate. However, the increase in 1-C lactate seems to indicate that ^13^C-labeled glucose has twice cycled the KC to become 1-C-pyruvate and then 1-C-lactate. This indicates that KC cycles much faster in SOD2 cells despite the low levels of SDH. This shortcut might be allowed by alanine-amino-transferase catalyzing the conversion from α t-ketoglutarate to pyruvate [29].

As we mentioned above, AMPK alteration might be responsible for this KC reprogramming; nevertheless, other scenarios are possible. The enzyme SDH is shared by KC and OXPHOS and presents a redox switch in six out of the nine Cys residues [30]. These two factors postulate SDH as a strategical sensor to transmit redox signals from the mitochondrial inner membrane, and therefore from OXPHOS, to the cytoplasm. We observed a clear decrease in Mitochondrial Complex II activity in SOD2-overexpressing cells. Succinate is considered an oncometabolite able to originate and promote malignant processes such as paraganglioma. Moreover, it also modulates the metabolic signature of tumor cells and their microenvironment [31]. Consequently, SDH is consider a tumor suppressor gene, and both succinate and SDH have been found upregulated and downregulated, respectively, in prostate cancer [32]. The alteration in SDH affecting both KC and Complex II from OXPHOS would form the third level of regulation exerted by SOD2. Apart from the decrease in Complex II, we could observe that the transit through the electron chain was increased, but this did not seem to affect ATP production, and the mitochondrial activity was decreased as indicated by the MTT assay.

The impact of SOD2 in cancer metabolism in general, and prostate cancer in particular, can be dramatic from a clinical perspective. *Sod2* polymorphisms are present in the population and the Ala16Val has a documented impact on SOD2 activity since the Val variant presents 30–40% less activity [33,34]. This variation of enzymatic activity is analogous to the one we have generated in our cell and mouse models. The Ala variant of this polymorphism has been consistently associated with prostate cancer risk and increased aggressiveness either alone or when the antioxidant intake is low [11,14,35,36,37], indicating that higher levels of SOD2 activity might help prostate cancer progression similarly to our results obtained in a study of a cohort of patients [12]. Moreover, redox-related metabolism can be induced by different environmental factors such as anti-tumor therapy, diet, or lifestyle factors [38]. For example, flavonoids and endogenous antioxidant derived from fruit and vegetables have been related to decreased risk of prostate cancer [15,39] and have a direct effect on GLUT-1 levels and glucose uptake. 

On the contrary, *Sod2* Val phenotype increases the risk of developing diabetes [40,41]. This may indicate that, supporting our results, in humans lower SOD2 activity decreases glucose uptake. Interestingly, diabetes is a protective factor for prostate cancer [5]. As for whether this protective effect is mediated by the process described here, the direct regulation of the GLUT-1 transporter by SOD2, this would need further studies. Those studies should be able to overcome the limitations of this study. 

First, our system recalls the variation observed due to Ala-polymorphism in terms of enzymatic activity (Enzymatic activity MOCK = 0.013 vs. SOD2 = 0.017; 30% increase). This causes metabolic features linked with tumor progression, such as amino-acid synthesis increase. However, whether the decrease can have a protective effect observed on the Val polymorphism should be elucidated. 

Second, there was a limited number of samples in our mice and patient cohorts. Although the results are robust especially for patient samples, a larger and more diverse cohort is needed to confirm our data. 

Third, performing the correspondent metabolic and metabolomic studies in in vivo models and patient samples could confirm that the plethora of effects observed with the *in vitro* model are also observed in mammals, beyond those glucose and GLUT-1 changes described in this manuscript. In the cell culture model, we observed a major decrease in SDH and a subsequent alteration of the KC; unfortunately, the limited access to samples did not allow further studies.

SOD2 is easily regulated by transcription factors during cancer progression [8], and also a polymorphism present in the population is able to decrease its activity by 30–40%. According to our results, the two situations described above may have an impact on glucose and metabolism in prostate cancer. These were, until now, unaccounted variables in disease management. It would be relevant whether SOD2 or H_2_O_2_ of endogenous source are increased in the prostate or if the patient is bearing any *Sod2* polymorphism, in order to predict therapy response or foreseeing the metabolic evolution. If redox metabolism is an easy-to-trigger switch that may in turn affect glucose and KC metabolism, it may happen often that the basal state differs substantially among patients. This could help to explain, for example, why a widespread tool for tumors follows up and relapses as 18F-glucose-PET shows such limited use in prostate cancer [42,43]. Metrics reporting the redox state, such as antioxidant capacity in serum, would be a very interesting tool. This, together with metabolic analytes, could be used for, for example, designing specific diets for prostate cancer patients, especially when they are undergoing anti-androgenic therapy. 

Under the light of these new results, we consider that SOD2 metabolism must be further studied in order to define its central role in prostate glucose metabolism, in order to improve management and treatment of prostate cancer.

## Data Availability

Data is available on University of Oviedo Repository. https://digibuo.uniovi.es/dspace/ (accessed on 10 December 2021).

## Supplementary Materials

The following are available online at https://www.mdpi.com/article/10.3390/antiox11020313/s1. Figure S1: Characterization of SOD2-overexpressing cells. Figure S2: Mitochondrial abundance in MOCK and SOD2 cells, GLUT-1 protein levels in SOD2 cells and GLUT-1 IHC positive control. Figure S3: Glucose Tolerance Test on WT and SOD2 transgenic mice females. Figure S4: SOD2 and GLUT-1 protein levels show a highly similar pattern in prostate adenocarcinoma patient samples. Table S1: Age and Gleason score of the prostate cancer patients (*n* = 5) used for this study. All patients had undergone radical prostatectomy without previous co-adjuvant therapy.
