# Supplementary material for: Androgen-Dependent Prostate Cancer Cells Reprogram Their Metabolic Signature upon GLUT1 Upregulation by Manganese Superoxide Dismutase
Source: Antioxidants (Basel). 2022 Feb 4;11(2):313. doi: 10.3390/antiox11020313 (PMC8868133; doi:10.3390/antiox11020313)
Supplement: Supplementary file 1 [file antioxidants-11-00313-s001.zip › antioxidants-1559713-supplementary.pdf]

Supplementary material

SUPPLEMENTARY MATERIAL AND METHODS

2.2. SOD 2 Enzymatic Activity

Reagents and sample preparation

Superoxide dismutase activity was determined with the xanthine-oxidase assay based on the oxidation of xanthine to produce superoxide anion which can oxidize cytochrome C. In the assay SOD can inhibit this reaction by removing anion superoxide. Xanthine (Sigma, X7375), xanthine oxidase (Sigma, X1875) and cytochrome c (Sigma, C2037) were used in the assay. Working xanthine solution 500 μM (from 0.3 M-NaOH stock) was diluted in PBS-0.1 Mm EDTA. Cytochrome C was dissolved in PBS-0,1 Mm EDTA to prepare a 100 μM solution. Final concentrations for the assay mixture: xanthine 50 μM, cytochrome c 10 μM and 0.1 mM EDTA. Xanthine oxidase solution was prepared apart with a concentration of 0.20 mU/πl in PB pH=7.8, 0.1 mM EDTA.

Cells were collected when they reached 75% confluency. Cell pellets resuspended in PBS were subjected to three freeze-thaw cycles to achieve the extraction of the native proteins and centrifuged at 3000 xg for 10 min at 4ºC.

To analyze specifically SOD2 activity, samples were treated with the cytosolic SOD inhibitor potassium cyanide (KCN) (Sigma, 60178) with a final concentration of 25 mM 20 min prior the assay.

2.13. Human prostate samples

Supplementary Table I: Age and Gleason score of the prostate cancer patients (n=5) used for this study. All of them undergone radical prostatectomy without previous co-adjuvant therapy.

ID	Gleason Score	NGS ISUP 2014*	Age
00_7011	4 (2+2)	1	62
00_7280	6 (3+3)	1	67
00_6143	5 (2+3)	1	60
08_20601	7 (3+4)	2	67
09_2326	9 (4+5)	9	55

* New Grading System (NGS) agreed during ISUP conference on Gleason grading of Prostatic Carcinoma

2.16. Mitochondria Complex II activity

Succinate dehydrogenase activity was measured according to (Assesment of mitochondrial respiratory chain enzymatic activities on tissues and cultured cells, M. Spinazzi 2012, Nat Prot) with minor volume modifications to adapt the assay to 96 well plates.

SUPPLEMENTARY FIGURES

SUPPLEMENTARY FIGURE 1

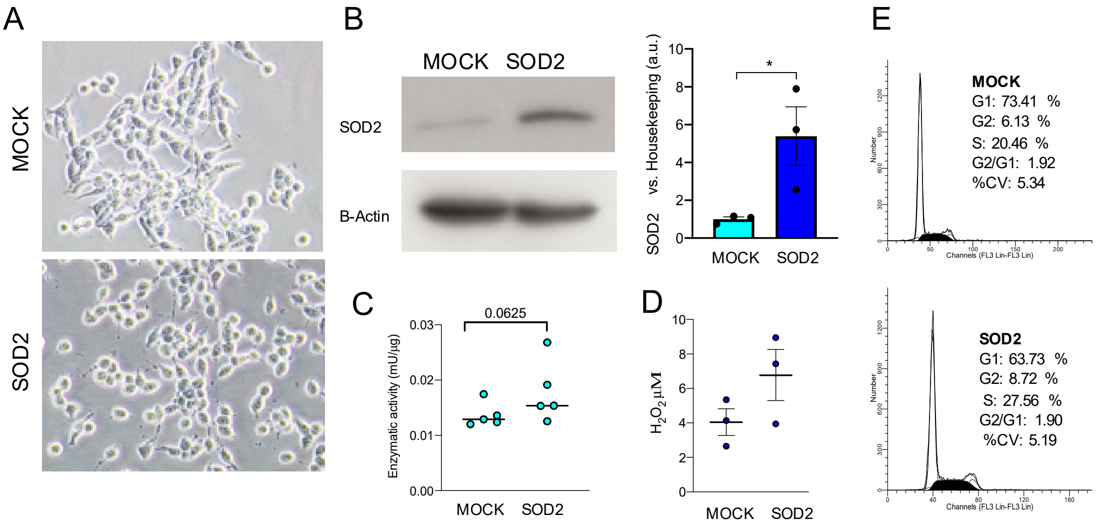

Supplementary figure 1: Characterization of SOD2 overexpressing cells. General features of LNCaP-Mock (MOCK) and LNCaP-SOD2 (SOD2) were studied. A) Micrograph showing general cell morphology (Magnification100x). B) Western-blot analysis of Sod2 protein levels including densitometric quantification (right). C) For the quantification of SOD2 enzymatic activity, SOD1/CuZnSOD was specifically inhibited by using KCN (25mM). D) Concentration of hydrogen peroxide quantified by electrochemistry in the extracellular media of MOCK and SOD2 cells. E) No significant differences in cell cycle phase distribution were observed between both cell types (n=3). For All panels, values are expressed as Mean±SEM; t test was used as statistical analysis; *= p-value<0.05, **=p-value<0.01, ***=p-value<0.001, ****=p-value<0.0001.

SUPPLEMENTARY FIGURE 2

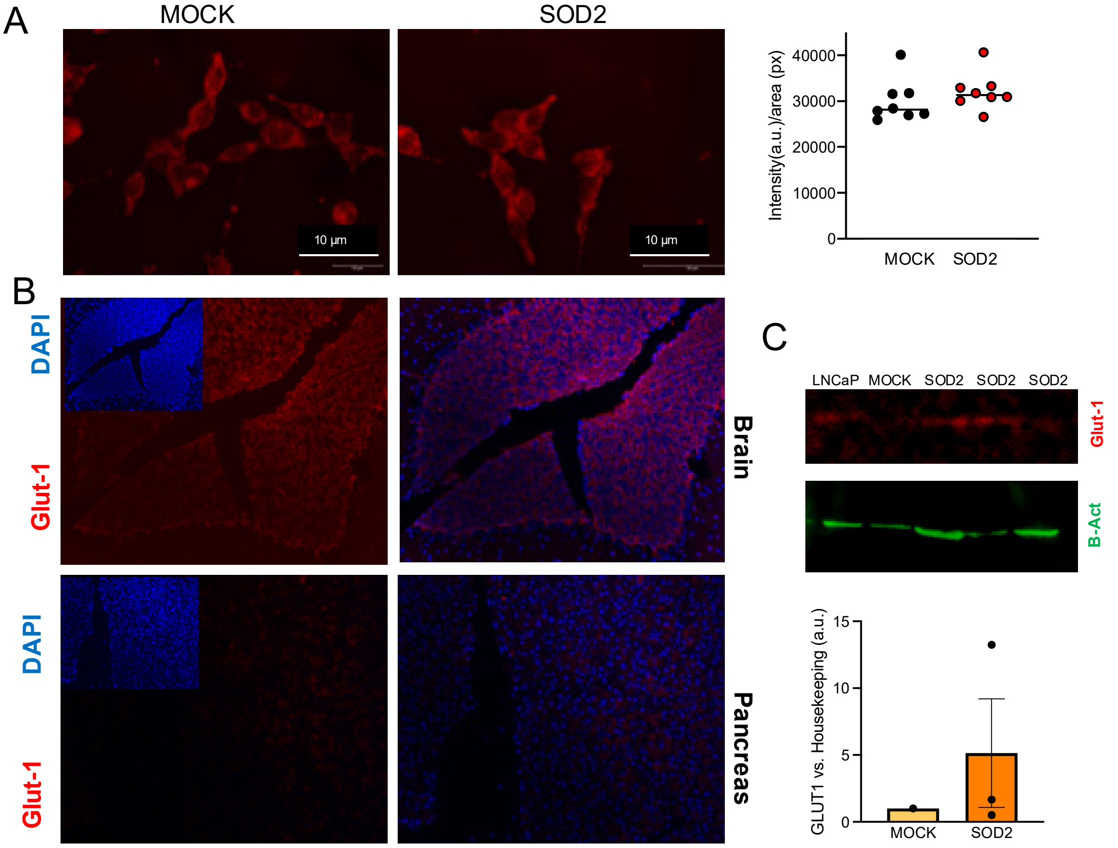

Supplementary figure 2: Mitochondrial abundance in MOCK and SOD2 cells and Glut-1 protein levels. A) Fluorescence mitochondrial probe MitoTracker^{TM} was used to determine average mitochondrial mass per cell. Individual R.O.I.s were drawn per cell in each field,e for each replicate (n=8), and average intensity per R.O.I. area was then calculated. B) Glut-1 antibody positive control. Brain (High protein levels) and Pancreas (Low protein levels) are shown, tissues were chosen according to Expression Atlas database (www.ebi.ac.uk). C) Western-blot analysis of Sod2 protein levels including densitometric quantification (below). Values are expressed as Mean±SEM.

SUPPLEMENTARY FIGURE 3

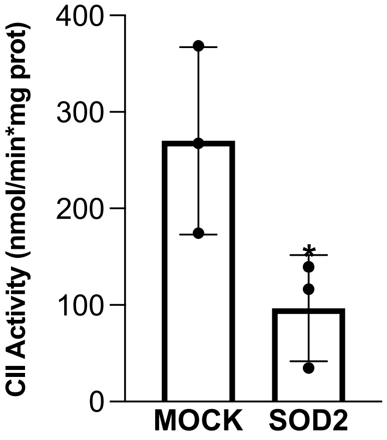

Supplementary figure 3: Mitochondrial Complex II activity MOCK and SOD2 cells. Complex II activity was measured following the protocol described by others (Spinazzi et al. 2012). Activity was evaluated by assessing the slope in presence or absence of inhibitor for both experimental groups. Values are expressed as Mean±SEM (n=3).

SUPPLEMENTARY FIGURE 4

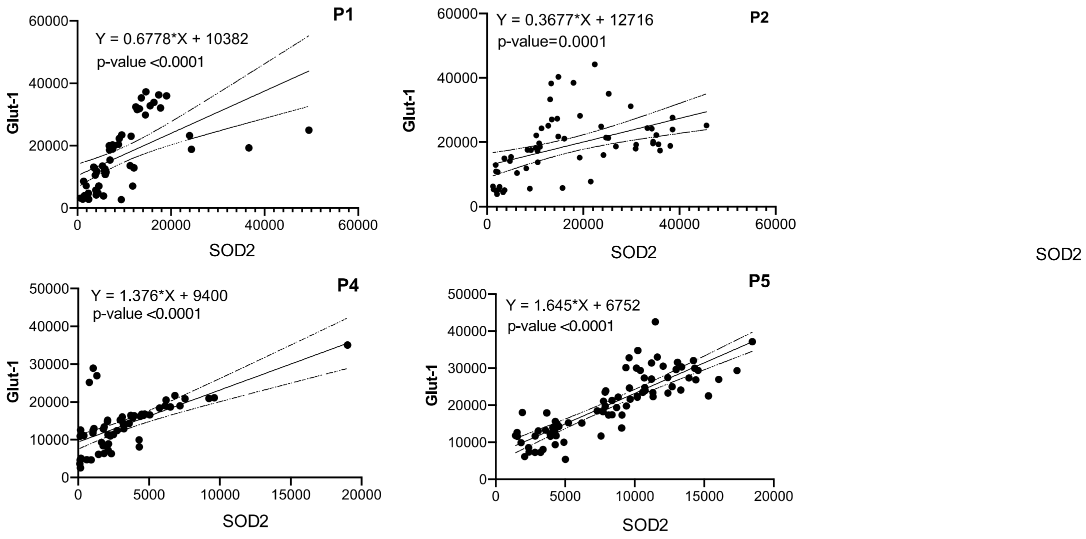

Supplementary figure 4: SOD2 and Glut-1 protein levels shows a highly similar pattern in prostate adenocarcinoma patient samples. Human prostate cancer samples (n=5) were analyzed for the expression pattern of SOD2 and Glut-1 proteins by using double IHF. Only glandular area was selected and the intensity of the signal for both proteins quantified. Correlation plot for each patient (P1-5) is show indicating single gland quantification values; correlation equations and p-values are shown.
